# Horizontal gene transfer contributes to virulence and antibiotic resistance of *Vibrio harveyi* 345 based on complete genome sequence analysis

**DOI:** 10.1186/s12864-019-6137-8

**Published:** 2019-10-22

**Authors:** Yiqin Deng, Haidong Xu, Youlu Su, Songlin Liu, Liwen Xu, Zhixun Guo, Jinjun Wu, Changhong Cheng, Juan Feng

**Affiliations:** 1 Key Laboratory of South China Sea Fishery Resources Exploitation & Utilization, Ministry of Agriculture and Rural Affairs, South China Sea Fisheries Research Institute, Chinese Academy of Fishery Sciences, Guangzhou, 510300 China; 2 Tropical Aquaculture Research and Development Centre, South China Sea Fisheries Research Institute, Chinese Academy of Fishery Sciences, Hainan, 572426 China; 3 Key Laboratory of Tropical Marine Bio-resources and Ecology, South China Sea Institute of Oceanology, Chinese Academy of Sciences, Guangzhou, 510301 China

**Keywords:** *Vibrio harveyi*, Virulence, Antibiotic resistance, Comparative genomics, Horizontal gene transfer

## Abstract

**Background:**

Horizontal gene transfer (HGT), which is affected by environmental pollution and climate change, promotes genetic communication, changing bacterial pathogenicity and drug resistance. However, few studies have been conducted on the effect of HGT on the high pathogenicity and drug resistance of the opportunistic pathogen *Vibrio harveyi*.

**Results:**

*V. harveyi* 345 that was multidrug resistant and infected *Epinephelus oanceolutus* was isolated from a diseased organism in Shenzhen, Southern China, an important and contaminated aquaculture area. Analysis of the entire genome sequence predicted 5678 genes including 487 virulence genes contributing to bacterial pathogenesis and 25 antibiotic-resistance genes (ARGs) contributing to antimicrobial resistance. Five ARGs (*tetm*, *tetb*, *qnrs*, *dfra17*, *and sul2*) and one virulence gene (CU052_28670) on the pAQU-type plasmid p345–185, provided direct evidence for HGT. Comparative genome analysis of 31 *V. harveyi* strains indicated that 217 genes and 7 gene families, including a class C beta-lactamase gene, a virulence-associated protein D gene, and an OmpA family protein gene were specific to strain *V. harveyi* 345. These genes could contribute to HGT or be horizontally transferred from other bacteria to enhance the virulence or antibiotic resistance of 345. Mobile genetic elements in 71 genomic islands encoding virulence factors for three type III secretion proteins and 13 type VI secretion system proteins, and two incomplete prophage sequences were detected that could be HGT transfer tools. Evaluation of the complete genome of *V. harveyi* 345 and comparative genomics indicated genomic exchange, especially exchange of pathogenic genes and drug-resistance genes by HGT contributing to pathogenicity and drug resistance. Climate change and continued environmental deterioration are expected to accelerate the HGT of *V. harveyi*, increasing its pathogenicity and drug resistance.

**Conclusion:**

This study provides timely information for further analysis of *V. harveyi* pathogenesis and antimicrobial resistance and developing pollution control measurements for coastal areas.

## Background

With the rapid expansion of aquaculture, vibriosis has become one of the most serious diseases to endanger sustainable aquaculture development [1, 2]. *Vibrio harveyi* is a halophilic, luminescent Gram-negative γ-proteobacteria, as an important pathogen of vibriosis [3–5]. *V. harveyi* has been extensively studied for more than a decade and is reported to be a serious pathogen for a range of marine vertebrates and invertebrates [4–7]. The earliest report of *V. harveyi* causing serious infections was to common snook (*Centropomus undecimalis*) in Florida, USA [8]. Subsequently, *V. harveyi* was shown to cause disease in *Penaeus monodon* and *Penaeus japonica*s in Thailand [10], brown-spotted grouper in Kuwait [9], common dentex (*Dentex dentex*) in Spain [10], *Holothuria scabra* (Holothuroidea, Echinodermata) in Toliara, Madagascar [11], Asian seabass (*Lates calcarifer*) in Malaysia [12], and *Epinephelus spp*. in China [13–15].

The wide use of antibiotics, increased environmental pollution and global climate change are leading to the enhancement of drug resistance of *Vibrio* spp., including *V. harveyi*. Several studies show that drug resistance and multidrug resistance (MDR) are occurring in aquaculture worldwide, including in shrimp ponds in Thailand, Malaysia, India, and China [16–19]; shellfish farms in Malaysia, Korea, USA, Poland and China [20–24]; and fish farms in Italy, Korea, and China [25–27]. Enhanced drug resistance leads to stronger virulence resulting in difficulty in preventing and treating *V. harveyi* infection [15, 16]. For example, Nakayama et al. [28] found that gradually increasing antibiotic concentration and frequent subculturing enhances *V. harveyi* antibiotic resistance, elevating the toxicity of *V. harveyi*. The pathogenic and drug-resistant genes of *V. harveyi* are a key to the fundamental cause of pathogenicity and drug resistance. Therefore, studying the pathogenic and drug-resistance genes of *V. harveyi* will provide an important foundation for determining pathogenic and drug-resistance mechanisms.

Although *V. harveyi* a recognized pathogen of marine animals, different strains vary in their ability to cause disease [29]. The main virulence factors of *V. harveyi* are extracellular proteases, outer membrane proteins, hemolysins, esterases, phospholipases, exotoxin and secretion systems [29, 30]. Production of antimicrobial enzymes that inactivate antibacterial drugs is an important resistant mechanism [31]. Inactivating enzymes can be expressed by genes on plasmids or chromosomes [32]. Virulence factors and antibiotic-resistance genes (ARGs) can also be vertically transferred and spread via horizontal gene transfer (HGT) through mobile genetic elements (MGEs) such as plasmids, bacteriophages, transposons, integrative and conjugative elements (ICEs), and genetic islands (GIs) [33]. Park et al. [34] found that MGEs that are prophages, GIs and pathogenicity islands carry different combinations of virulence factors that promote immune evasion and superantigens that contribute to serious *Staphylococcus aureus* infection. Le et al. [35] reported that 0.92% (36 of 38,895) analyzed proteins from 31 *Rickettsiales* genomes are associated with strong bootstrap support for HGT with function as ATPases, aldolases, transporter activities, cystathionine beta-lyases, sugar phosphate permeases, growth inhibitors and antitoxin activities. For horizontal transfer, exogenous DNA must evade the bacterial immune system such as restriction modification systems (RM systems) and CRISPR-Cas systems [36, 37].

Serious infection and multidrug resistance lead to the emergency of the availability of whole genome sequences for different *V. harveyi* strains to determine pathogenic and antimicrobial-resistance mechanisms. Evidence is growing that HGT is an important driving force for prokaryotic evolution affecting pathogenicity and drug resistance [34, 38]. Coastal urbanization has recently intensified, resulting in the production of a large amount of antibiotics, heavy metals, and nutrients pollutants (Additional file 1: Table S1), which may increase the pathogenicity and drug resistance of *V. harveyi* by affecting HGT [39, 40]. *V. harveyi* is the dominant species that causes serious infection and mortality of farmed fish in Guangdong, Southern China [14, 15]. However, little information is available on the mechanism of *V. harveyi* pathogenesis and drug resistance.

The predominant strain *V. harveyi* 345 is multidrug resistant to ampicillin, rifampicin, tetracycline, pediatric compound sulfamethoxazole tablets, vancomycin, doxycycline, trimethoprim, streptomycin, kanamycin, sulfamethoxazole, furazolidone, cefixime, and chloramphenicol. This strain was isolated from a diseased *E. oanceolutus* in Shenzhen, Southern China. *V. harveyi* 345 is suspected to lead to kidney enlargement and softening, spleen enlargement, and anal bleeding of *E. oanceolutus* and has a median lethal dose (LD_50_) of 9.83 × 10^5^ CFU·g^− 1^ [41]. Therefore, its complete genome information and HGT events are helpful for clarifying *V. harveyi* pathogenesis and drug resistance, controlling *V. harveyi* disease and reducing economic losses. In this study, we present the entire genome sequence of *V. harveyi* 345 with comparative genomics analysis of its pathogenesis, antimicrobial resistance and genome expansion caused by HGT.

## Results

### General features of *V. harveyi* 345

**Fig. 1:**
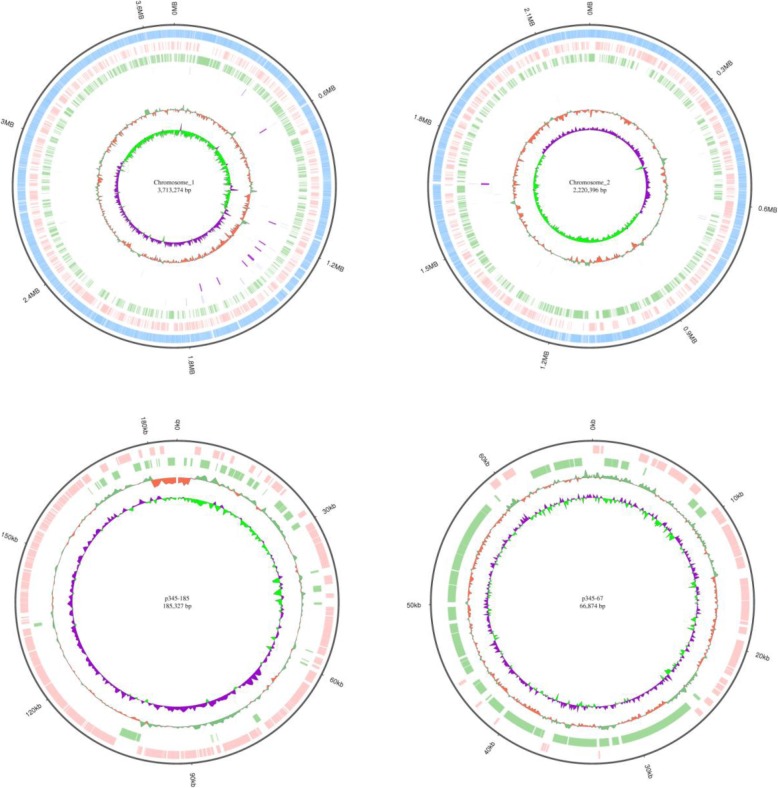
Circular representation of the *V. harveyi* 345 genome. Inner to outer: genome size, all annotated genes, forward-strand genes, reverse-strand genes, tRNA, rRNA, sRNA, GC and GC-skew

A total of 1189 megabases clean data and 98,602 subreads were generated assembling into two contigs with average genome coverage of 41.71×. After error correction, GATK analysis and gap filling, a genome of 6,185,822 bp was generated, which was smaller than comparable strains KC13.17.5, E385, and 74F and larger than 27 other comparable strains (Additional file 2: Table S2). The chromosomal G + C content (44.76%) of 345 agreed with values from 30 comparable strains (44.60–45.60%) (Additional file 2: Table S2). Combined sequencing analysis revealed that the complete genome of *V. harveyi* 345 contained two circular chromosomes, named ChI (3,713,225 bp, 44.81% G + C, CP025537) and ChII (2,220,396 bp, 44.81% G + C, CP025538), and two circular megaplasmids named p345–185 (185,327 bp, 43.48% G + C, CP025539) and p345–67 (66,874 bp, 43.48% G + C, CP025540) (Table 1 and Fig. 1).

**Table 1:** Genome features of *V. harveyi* 345

Features	ChI	ChII	Plasmid p345–185	Plasmid p345–67
Length (bp)	3,713,225	2,220,396	185,327	66,874
G + C content (%)	44.81	44.81	43.48	43.48
Genes	3345	2044	208	81
rRNA gene	28	3	0	0
tRNA genes	113	15	0	0
sRNA genes	6	7	0	0
Minisatellite DNA	26	15	2	1
Microsatellite DNA	22	11	1	1
GenBank accession no.	CP025537	CP025538	CP025539	CP025540

### Gene annotation

**Fig. 2:**
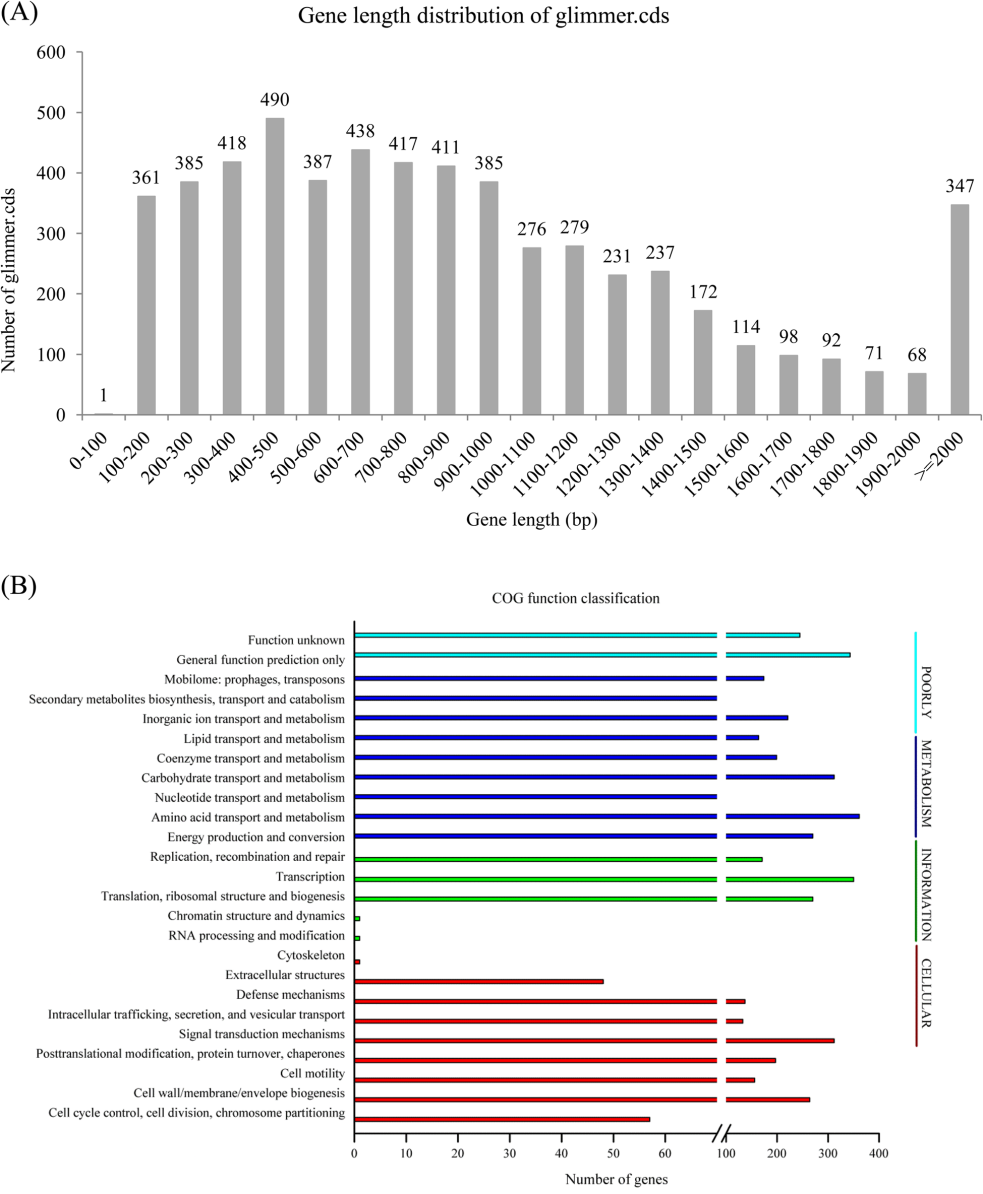
Coding sequence length distribution and COG functional classifications. Coding sequence length distribution of *V. harveyi* 345 (**a**). COG functional classifications of *V. harveyi* 345 coding sequences (**b**)

A total of 5678 genes (fewer than ZJ0603 and more than 29 other strains) (Additional file 2: Table S2) were predicted to be an average 929 bp with a mean GC content of 45.68%, accounting for 85.29% of the genome. The three highest levels of gene length distribution were 400–500 bp, 600–700 bp, and 300–400 bp and the three lowest were 0–100 bp, 1900–2000 bp, and 1800–1900 bp (Fig. 2a). In addition, 128 tRNA, 31 rRNA and 13 sRNA genes were identified with confidence. A total of 89 tandem repeats, including 44 minisatellite DNA and 35 microsatellite DNA, were predicted (Table 1 and Fig. 1).

Predicted open reading frames (ORFs) were further classified into Clusters of Orthologous Groups (COG) (4586 ORFs in total contenting 80.77%, Fig. 2b). According to COG categorization analysis, the top five groups in abundance were amino acid transport and metabolism (361 ORFs), transcription (350 ORFs), general function prediction only (343 ORFs), signal transduction mechanisms (312 ORFs) and carbohydrate transport and metabolism (312 ORFs) (Fig. 2b). In addition, 3247 genes were classified into 40 functional Kyoto Encyclopedia of Genes and Genomes (KEGG) pathways (Additional file 3: Figure S1). Some genes were involved in more than one KEGG pathway and included the top five pathways for replication and repair (784 ORFs), carbohydrate metabolism (633 ORFs), signal transduction (506 ORFs), infectious diseases (463 ORFs) and membrane transport (432 ORFs). Furthermore, 3453 genes were classified into 40 functional Gene Ontology (GO) classifications (Additional file 4: Figure S2). Some genes were involved in more than one GO classifications and included the top five classifications of metabolic process (1914 ORFs), cellular process (1881 ORFs), catalytic activity (1785 ORFs), single-organism process (1526 ORFs) and binding (1494 ORFs).

Twelve RM system genes were annotated in *V. harveyi* strain 345 (Table 2). Ten belonged to Type I RM systems, encoding three R subunits (*hsdR*), two S subunits (*hsdS*) and five M subunits (*hsdM*). The other two belonged to type III RM systems, encoding a methyltransferase (*mod*) and a restriction enzyme (*res*).

**Table 2:** The RM system of *V. harveyi* 345

Gene_id	Gene name	Products
CU052_00675	*hsdR*	type I restriction enzyme, R subunit
CU052_10560	*hsdR*	type I restriction enzyme, R subunit
CU052_10570	*hsdS*	type I restriction enzyme, S subunit
CU052_10575	*hsdM*	type I restriction enzyme, M protein
CU052_17245	*hsdM*	type I restriction enzyme, M protein
CU052_17265	*hsdR*	type I restriction enzyme, R subunit
CU052_17270	*hsdS*	type I restriction enzyme, S subunit
CU052_17275	*hsdM*	type I restriction enzyme, M protein
CU052_17285	*hsdM*	type I restriction enzyme, M protein
CU052_27190	*hsdM*	type I restriction enzyme, M protein
CU052_16835	*mod*	adenine-specific DNA-methyltransferase
CU052_16840	*res*	type III restriction enzyme

### Virulence factors

A total of 487 putative virulence factors were predicted (Table 3 and Additional file 5: Table S3), involving adherence (type IV pili, lipooligosaccharide LOS, and OmpU), motility (polar flagellar proteins, chemotaxis proteins, lateral flagellin proteins, and motor proteins), regulation (Autoinducer-2 and cholerae autoinducer-1), secretion system (type II/III/IV/VI secretion system proteins, T2/3/4/6SS proteins), and iron uptake. Many genes were identical to genera other than *Vibrio* such as *Escherichia*, *Pseudomonas*, *Salmonella*, *Aeromonas*, and *Mycobacterium*. T3SS1, which is widely reported to be involved in *Vibrio* pathogenesis, was found on ChI. T6SSs are implicated in cell targeting and virulence, and are believed to mediate antibacteria [42]. Six T6SS proteins on Hcp secretion island-1 were detected. The *tlh* gene encoding a thermolabile hemolysin (TLH) was on ChII. In addition, four virulence genes (CU052_28670, CU052_00025, CU052_00190, and CU052_00400) were on plasmid with CU052_28670 on p345–185, and CU052_00025, CU052_00190, and CU052_00400 on p345–67.

**Table 3:** Virulence factors predicted with VFDB database

Virulence factor	Annotation	Chromosome	Identity genus
Adherence			
*acfA*	ACF	II	*Vibrio*
*IlpA*	IlpA	I	*Vibrio*
*VP1611*	MAM7	I	*Vibrio*
*mshH, mshI, mshI, mshJ, mshK, mshL, mshM, mshN, mshE,*	MSHA type IV pilus	I, II	*Vibrio*
*mshG, mshF, mshA, mshD, mshA, mshA, mshA, mshA,*			
*ompU*	OmpU	I	*Vibrio*
*aatC, aatC*	TCP	II	*Escherichia*
*flp-1, flp-1,tadB, tadC, tadA, flp, rcpC, rcpA, tadZ, tadA,*	Flp type IV pili	I, II	*Vibrio, Aggregatibacter*
*tadB, tadC, tadD, tadE, tadF, tadG, rcpA, tadA*			
*vfr, rpoN, rpoS, pilW, fimT, pilE, pilM, pilN, pilO, pilP,*	type IV pili	I, II	*Vibrio, Pseudomonas, Salmonella,*
*pilQ, pilT, pilU, pilF, pilT, pilR, pilR, pilR, pilW, pilA,*			*Dichelobacter, Yersinia, Haemophilus*
*pilR, pilR, pil, R, pilD, pilC, pilB*			
*CJJ81176_1161, CFF8240_1412, CFF8240_1412,*	LOS	I, II	*Campylobacter, Haemophilus*
*C8J_1080, kdsB, lpxK, msbA, kdsA, lpxC, gmhA/lpcA,*			
*htrB, lgtF, lgtF,opsX/rfaC, kdkA,msbB, rfaD, orfM, htrB,*			
*galE, lpxA, lpxB,gmhA/lpcA, htrB, lpxH, yhxB/manB,*			
*galE, msbA, rfaE, neuC1, lpxD*			
*tuf, tuf*	EF-Tu	I	*Mycoplasma*
*htpB*	Hsp60	I	*Legionella*
*lap*	Listeria adhesion protein	I	*Listeria*
*CT396, CT396*	MOMP	I	*Chlamydia*
Motility			
*flaD, flaA, flaL, flaK, flaJ, flaI, flaH, flaG, flaF, flaE,*	Flagella	I, II	*Vibrio, Pseudomonas, Salmonella,*
*flaD, flaC, flaB, cheR, cheA, cheB, cheV, cheV, cheW,*			*Legionella, Aeromonas, Pseudomonas*
*cheW, cheY, cheZ, filM,flaB, flaD, flaD, flaE, flaG, flaI,*			
*fleQ, fleQ, fleR, flgA, flgA, flgB, flgC, flgD, flgE,*			
*flgF, flgG, flgH, flgI, flgJ, flgK, flgL, flgM, flgM flgN,*			
*flgN, flgO, flgP, flgT, flhA, flhA, flhB, flhB, flhF, flhG,*			
*fliA, fliD, fliE, fliE, fliF, fliF, fliG, fliG, fliH, fliH, fliI,*			
*fliI, fliJ, fliJ, fliK, fliL, fliM, fliN, fliN, fliO, fliP, fliP,*			
*fliQ, fliQ, fliR, fliR, fliS, fliY, flmH, flmH, flmH, flrA,*			
*flrB, flrC, lafA, lafB, lafC, lafD, lafE, lafF, lafK,*			
*lafS, lafU/motB, lafW, motA, motA, motB, motX, motY,*			
*motY, scrA, scrB, scrC, scrG, B565_1123*			
Regulation			
*luxS, adeG*	AI-2	I, II	*Vibrio, Acinetobacter*
*cqsA*	CAI-1	II	*Vibrio*
Secretion system			
*epsA, epsB, epsC, epsE, epsF, epsG, epsH, epsI, epsJ,*	EPS T2SS	I	*Vibrio*
*epsK, epsL, epsM, epsN, gspD*			
*exsA, exsD, sycN, tyeA, vcrD, vcrG, vcrH, vcrR, vcrV,*	T3SS1	I	*Vibrio*
*vecA, virG, vopB, vopD, vopN, vopQ, vopR, vscB, vscC,*			
*vscD, vscF, vscG, vscH, vscI, vscJ, vscK, vscL, vscN,*			
*vscO, vscQ, vscR, vscS, vscT, vsxU, vscX, vscY, vscC*			
*hopAJ2, hopJ1, mlr6326, ati2, ati1*	Other T3SS	I, II	*Pseudomonas, Mesorhizobium, Aeromonas*
*lpg2936, trwD, virB1, virB10, CbuK_1823, CbuG_0575,*	T4SS	I, II	*Legionella, Bartonella, Brucella, Coxiella*
*CbuG_1738, COXBURSA331_A0369*			
*PFLU6009, Psyr_2628, clpV1, pppA, clpV1, Psyr_2628,*	T6SS	I, II	*Pseudomonas, Burkholderia, Citrobacter,*
*tssH/clpV/bscF, cts2A, lip2, impB, impB, impC, impC*			*Agrobacterium*
Toxin			
*secA2*	Ace	I	*Mycobacterium*
*tlh*	Thermolabile hemolysin	II	*Vibrio* (*Vibrio parahaemolyticus*)
*clbQ, clbM, clbL, clbI, clbH, clbF, clbE, clbD, clbC*	Colibactin	I	*Escherichia*
*vctA*	Enterobactin	II	*Vibrio*
*ast*	Enterotoxin	I	*Aeromonas*
Immune evasion			
*ABK1_0097, ABK1_0097, BC5270, BC5275, BC5275,*	Capsule	I, II	*Vibrio, Staphylococcus, Mycoplasma,*
*BCE_5384, cap8J, cpsA, cpsB, cpsB, cpsC, cpsD, cpsE,*			*Enterococcus, Acinetobacter, Neisseria,*
*cpsF, cpsG, cpsJ, eps9, eps9, kpsF, kpsF, oppF, oppF,*			*Enterococcus, Campylobacter, Burkholderia,*
*oppF, rmlB, rmlD, sicC/synD, uppS, wbfB, wbfV/wcvB,*			*Bacillus*
*wcbN, wecA, wza, wzb, wzc*			
*fabZ, acpXL, kbtB, waaZ, waaF, gtrB*	LPS	I	*Brucella, Helicobacter, Pseudomonas, Shigella*
*ACICU_00876, ASA_0824, ASA_3329, cbsB, ccmA, ccmB,*	Iron uptake	I, II	*Vibrio, Acinetobacter, Aeromonas,*
*ccmC,ccmE, ccmF, ccmF, chuY, ciuD, fur, hemA, hemB,*			*Burkholderia, Dickeya, Escherichia,*
*hemC, hemE, hemG, hemH, hemL, hemN, hemN, hitA, hitB,*			*Haemophilus, Legionella, Mycobacterium,*
*hitC, hitC, hmuU, hutA, huvB, huvC, huvD, huvX, huvZ,*			*Pseudomonas, Salmonella, Shigella,*
*iutA, mbtI, orbG, orbH, pchH, phuV, PSEEN2499, psuA, pvdH,*			*Yersinia*
*pvsA, pvsB, pvsC, pvsD, pvsE, pvuA, pvuB, pvuC, pvuD,*			
*pvuE, sitA, sitB, sitC, sitD, vabC, vctC, vctC, vctD, vctD,*			
*vctG, vctG, vctP, viuC*			
Antiphagocytosis			
*algB, algW, algW, algU, mucP, algR*	Alginate	I, II	*Pseudomonas*
*pgi, mrsA/glmM*	Exopolysaccharide	I	*Haemophilus*
*hasD, hasB, hasF*	Hyaluronic acid capsule	I, II	*Pseudomonas, Serratia, Yersinia*
*katA, katA, clpC, clpE, clpP, ctpV, ibpA, msrA/B (pilB),*	Stress protein	I, II	*Neisseria, Listeria, Mycobacterium,*
*regX3, regX3, sodB, sodB, sodC1*			*Legionella, Salmonel*la
Exoenzyme			
*icl*	Isocitratase	I	*Mycobacterium*
*mip, mip*	Mip	I, II	*Legionella*
*Regulation*			*Mycobacterium, Legionella, Salmonella*
*mprA, mprA, mprA, relA, rpoS, csrA, sigA*	Regulation	I	
Efflux pump			
*mtrD*	MtrCDE	I	*Neisseria*
*panC*	Metabolic adaptation		*Mycobacterium*
Others			
*csgD, csgD, glnA1, ML1683, ML1683, leuD, MGA_1142,*	Others		
*ipaH, ECS88_3547, ndk, pdhB, ppsE, cysC1, argK, eno, plr/gapA,*		I, II	*Vibrio, Acinetobacter, Escherichia,*
*hpt, sugC, sugC, sugC, sugC, sugC, trpD, bfmR, bfmR, bfmR, bfmR,*			*Listeria, Mycobacterium, Mycoplasma*,
*vibD, vibE, vibA, VVA1298, VVA1298, VVA1298, vctP*			*Pasteurella, Pseudomonas, Salmonella,*
			*Shigella, Streptococcus*

### Antimicrobial-resistance genes

We identified 25 genes predicted to have > 40% identity to well-characterized ARGs (Table 4), including genes for aminoglycoside (*golc*, *adeb*, *emre*), penicillin (*pbp2*, *pbp1a*, *pbp1b*, *pbp2*), tetracycline (*tet34*, *tetm*, *tetb*), chloramphenicol (*adeb*, *ceob*, *mdtl*) and trimethoprim resistance (*dfra26*). In addition, five genes (CU052_28095, CU052_28120, CU052_28525, CU052_28540, and CU052_29140) were on the plasmid p345–185. These results were consistent with antibiotic-susceptibility assay data for strain 345 showing resistance to streptomycin, ampicillin, tetracycline, chloramphenicol, trimethoprim, and sulfamethoxazole.

**Table 4:** Antimicrobial resistance genes predicted with the ARDB database

Gene_id	Identity (%)	Resistance_Type	Antibiotic_Resistance	Description
CU052_02205	47.53	*pbp2*	penicillin	penicillin-binding protein 2
CU052_02455	99.35	*tet34*	tetracycline	oxytetracycline resistance phosphoribosyltransferase domain-containing protein Tet(34)
CU052_03895	47.65	*tolc*	aminoglycoside, glycylcycline, macrolide, beta_lactam,acriflavin	outer membrane channel protein TolC
CU052_04185	67.79	*ksga*	kasugamycin	16S rRNA (adenine(1518)-N(6)/adenine(1519)-N(6))-dimethyltransferase RsmA
CU052_04200	40.24	*dfra26*	trimethoprim	type 3 dihydrofolate reductase
CU052_05755	49.16	*mexw*	–	multidrug efflux RND transporter permease subunit
CU052_07910	46.51	*pbp1a*	penicillin	PBP1A family penicillin-binding protein
CU052_09355	42.63	*pbp1b*	penicillin	penicillin-binding protein 1B
CU052_09890	49.11	*pbp2*	penicillin	penicillin-binding protein 2
CU052_13950	51.93	*adeb*	chloramphenicol, aminoglycoside	multidrug efflux RND transporter permease subunit
CU052_15205	98.25	*norm*	tigecycline, streptomycin, kanamycin, ciprofloxacin, norfloxacin	MATE family efflux transporter
CU052_15655	43.59	*emre*	aminoglycoside	QacE family quaternary ammonium compound efflux SMR transporter
CU052_16225	48.04	*bcr*	–	Bcr/CflA family drug resistance efflux transporter
CU052_21770	40.74	*ykkc*	na_antimicrobials	QacE family quaternary ammonium compound efflux SMR transporter
CU052_23115	43.06	*ceob*	chloramphenicol	multidrug efflux RND transporter permease subunit
CU052_25305	44.41	*emrd*	–	multidrug transporter EmrD
CU052_26580	43.94	*catb5*	chloramphenicol	antibiotic acetyltransferase
CU052_27055	58.33	*qnra*	fluoroquinolone	Qnr family pentapeptide repeat protein
CU052_27855	62.23	*mdtl*	chloramphenicol	multidrug transporter MdtL
CU052_28095	100	*tetm*	tetracycline	tetracycline resistance ribosomal protection protein Tet(M)
CU052_28120	100	*tetb*	tetracycline	tetracycline efflux MFS transporter Tet(B)
CU052_28525	67.43	*qnrs*	fluoroquinolone	quinolone resistance pentapeptide repeat protein QnrVC6
CU052_28540	64.47	*dfra17*	Trimethoprim	trimethoprim-resistant dihydrofolate reductase DfrA
CU052_29140	100	*sul2*	sulfonamide	sulfonamide-resistant dihydropteroate synthase Sul2

### Genomic islands, prophages and CRISPR-Cas systems

Forty-seven GIs were detected on ChI (Additional file 6: Table S4) and 24 on ChII in *V. harveyi* 345 (Additional file 7: Table S5). In the GIs of ChI, 40 transposases were encoded belonging to the IS110, IS21, IS256, IS3, IS5/IS1182, IS66, IS91, ISL3, and ISNCY families and seven belonging to two GIs. Another eight genes were identified that encoded IS66 family insertion sequence hypothetical proteins. Three integrases and one serine recombinase were encoded. Three genes were predicted to encode type III secretion proteins and one was predicted to encode another virulence factor (CU052_RS12915). In the GIs of ChII, nine genes encoding transposases belonging to the IS3 and ISNCY families were detected with four belonging to more than one GI. We found three genes encoding integrases and 13 genes on three GIs predicted to encode type VI secretion system (T6SS) proteins.

Two intact prophage sequences were identified (Additional file 8: Table S6 and no CRISPR elements were predicted.

### Phylogenetic analysis, pan-core genes, dispensable and strain-specific genes

**Fig. 3:**
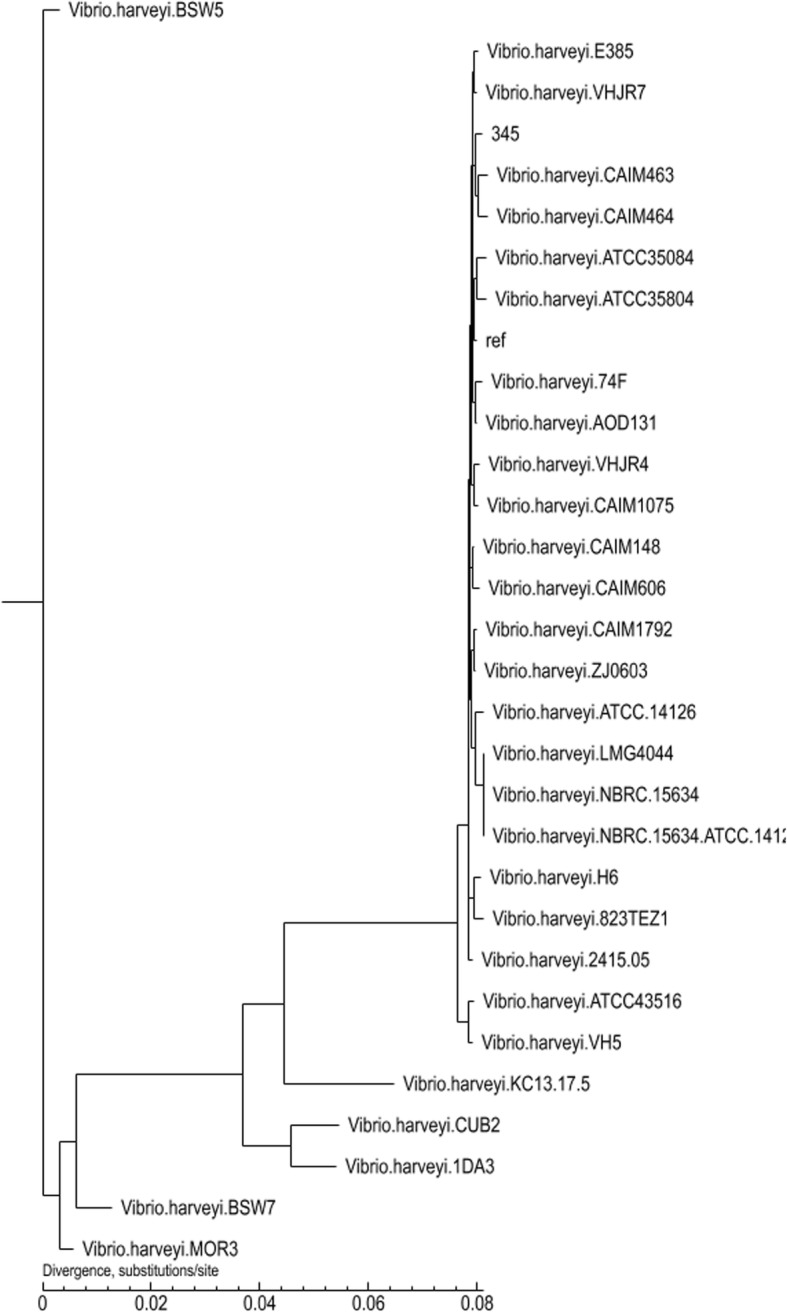
Phylogenetic relationship of *V. harveyi* 345 and 30 compared *V. harveyi* strains. Results are based on the SNP matrix of the 31 strains and Treebest-1.9.2 software and maximum-likelihood method

Phylogenetic trees showed that *V. harveyi* 345 closely related to other *V. harveyi* strains, especially *V. harveyi* VHJR7 and *V. harveyi* CAIM463 (Fig. 3)*.*

**Fig. 4:**
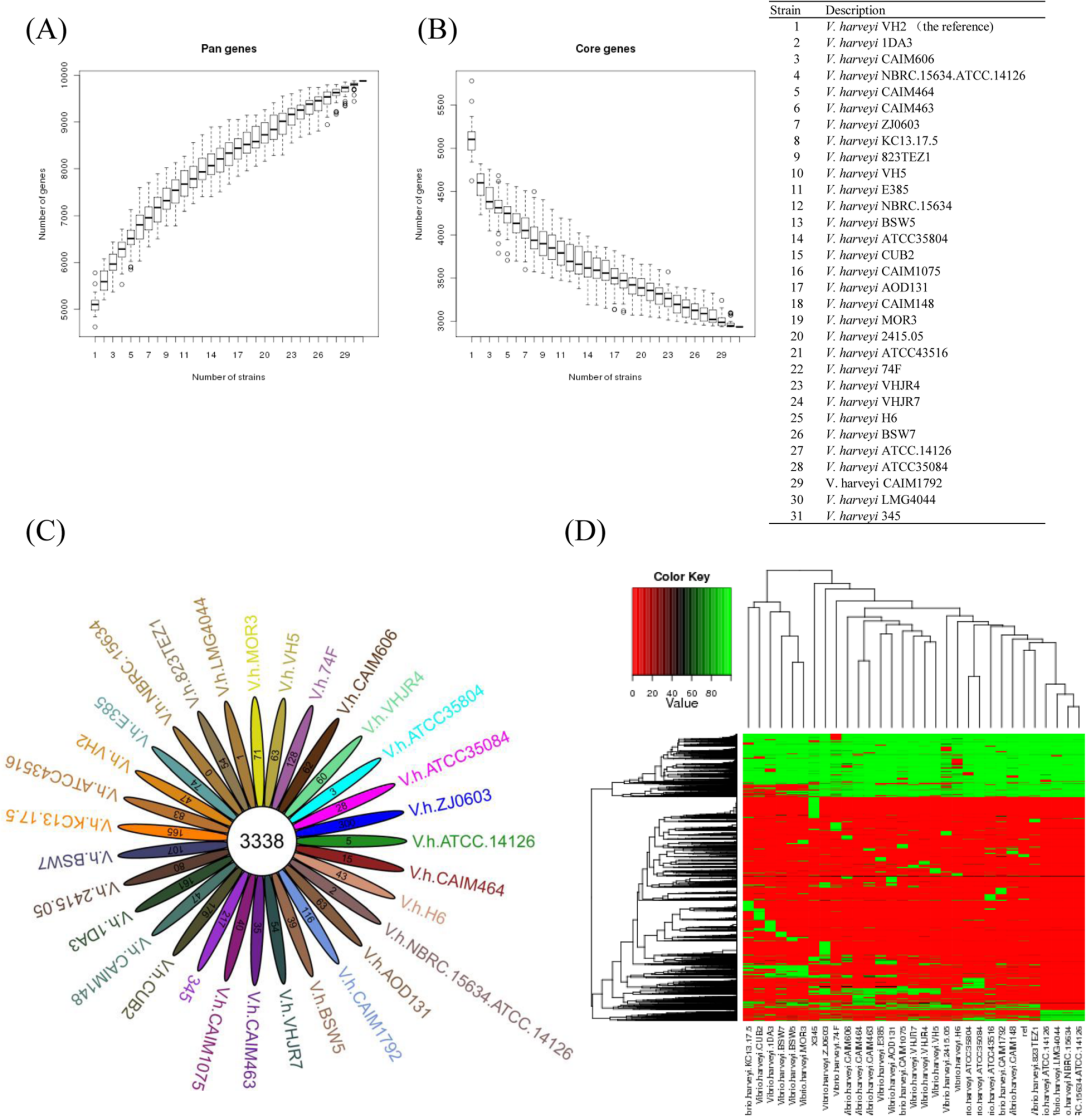
Pan-core genes, strain-specific and dispensable genes. Pan-gene dilution curve of 31 *V. harveyi* strains (**a**), core-gene dilution curve of the 31 strains (**b**), flower plot of strain-specific genes and core-genes (**c**), and heat map after removal of core-gene (dispensable heatmap) (**d**)

**Fig. 5:**
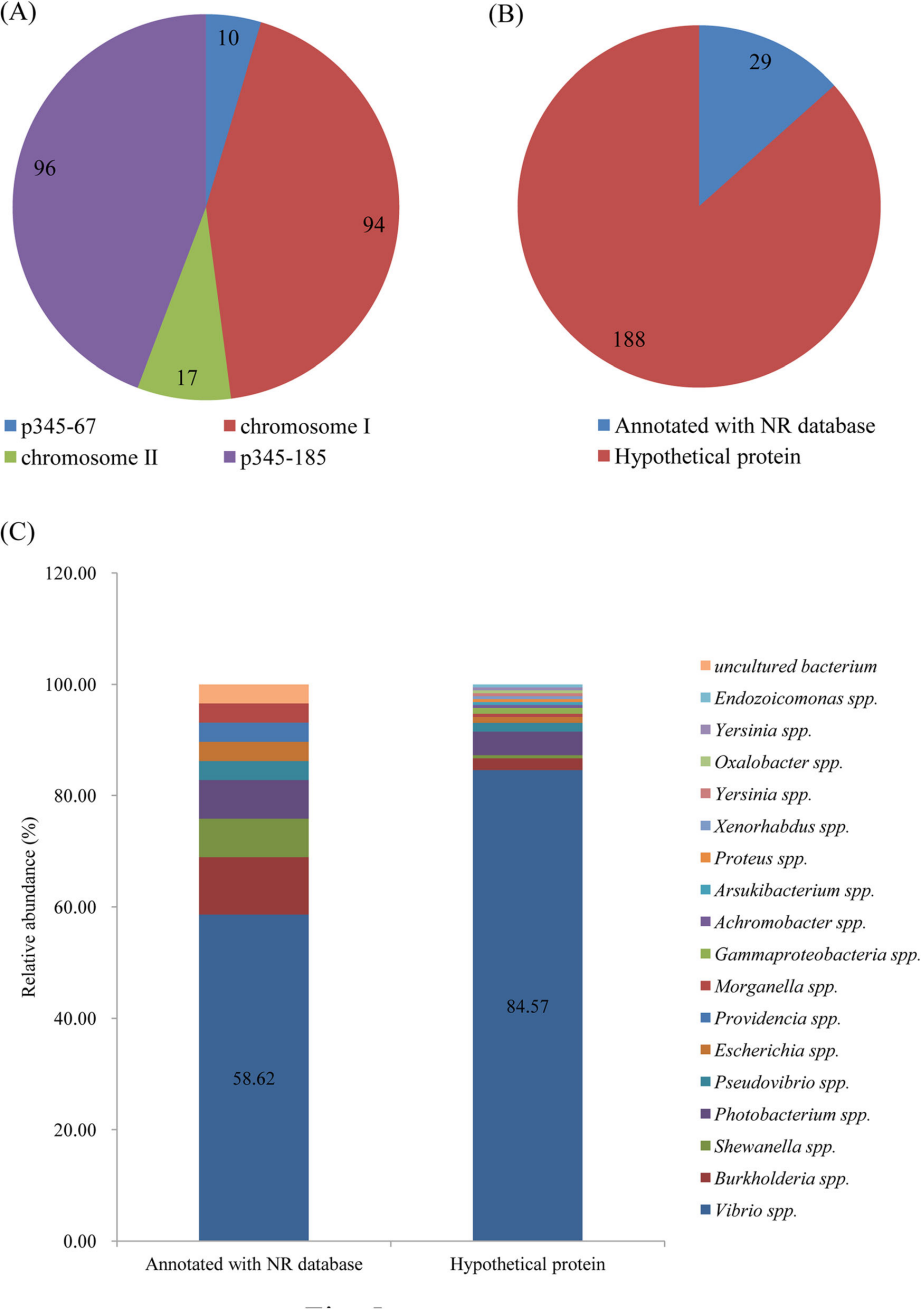
Strain-specific genes of *V. harveyi* 345. Strain-specific genes locations (**a**). Gene number of hypothetical proteins and proteins annotated by NR Database (**b**). Composition and relative abundance of strain-specific genes (**c**)

The pan-gene refers to the total number of genes in all 31 strains, while core-gene represents orthologous genes shared among them. Dispensable genes were not included in at least one of the 31 strains and strain-specific genes were included in only one strain. Using 31 genomes (Additional file 2: Table S2), 9878 pan-genes with a total size of 2,764,797 amino acid (aa) and 3338 core-genes with a total size of 1,133,465 aa were identified (Fig. 4a-b-c). A dispensable gene heatmap showed that each strain contained strain-specific genes (Fig. 4d). A total of 217 genes were specific to *V. harveyi* 345 with 10 on p345–67, 94 on ChI, 17 on ChII, and 96 on p345–185 (Additional file 9: Table S7, Fig. 5a). Twenty-nine genes were annotated by Non-Redundant (NR) database and the others were hypothetical protein-encoding genes (Additional file 9: Table S7, Fig. 5b). Four annotated genes encoded MGEs, including two type IV conjugative transfer proteins (CU052_28715 and CU052_28750) and two recombinases (CU052_29120 and CU052_00050) on p345–185 or p345–67. Four (CU052_13455, CU052_13535, CU052_13525, and CU052_13445) were phage-related genes on ChI. Four genes (CU052_14535, CU052_14240, CU052_23640, and CU052_13020) on Chr I encoded membrane proteins. One beta-lactamase class C-encoding gene (CU052_00920) was on Chr I. Two DNA methylase-encoding genes (CU052_28385 and CU052_28885) belonging to the bacterial immune system were on p345–185. One virulence-associated protein D (CU052_13320) was on Chr I (Additional file 9: Table S7).

### Strain-specific gene family

A strain**-**specific gene family was present in one of the 31 strains. Seven gene families were strain specific to *V. harveyi* 345 with > 50% identity to *Pseudovibrio* spp., *Photobacterium* spp., *Escherichia* spp., and other *Vibrio* spp. (*V. parahaemolyticus*) and were involved in recombination, peptide synthesis, conjugation and integration (Table 5).

**Table 5:** Strain-specific gene families of *V. harveyi* 345

Gene Family ID	Locus	Identity	E_value	Subject ID	Subject_description
1	CU052_06110	91.98	1.00E-125	gi|752,301,544|ref.|WP_041157164.1|	hypothetical protein, partial [*Vibrio mytili*]
1	CU052_29120	100	0.00E+ 00	gi|446,775,679|ref.|WP_000852935.1|	recombinase [*Escherichia coli*]
2	CU052_00970	62	0.00E+ 00	gi|359,344,462|gb|AEV37836.1|	Putative peptide synthetase [*Pseudovibrio* sp. FO-BEG1]
2	CU052_00980	55.33	0.00E+ 00	gi|504,051,870|ref.|WP_014285864.1|	non-ribosomal peptide synthetase [*Pseudovibrio* sp. FO-BEG1]
3	CU052_00975	61.49	2.00E-125	gi|504,051,870|ref.|WP_014285864.1|	non-ribosomal peptide synthetase [*Pseudovibrio* sp. FO-BEG1]
3	CU052_00965	62.42	0.00E+ 00	gi|359,344,462|gb|AEV37836.1|	Putative peptide synthetase [*Pseudovibrio *sp. FO-BEG1]
4	CU052_28750	100	8.00E-84	gi|760,459,713|gb|AJP18243.1|	MULTISPECIES: type IV conjugative transfer system protein TraA [*Vibrio parahaemolyticus*]
4	CU052_28755	100	3.00E-84	gi|691,549,048|ref.|WP_032073022.1|	hypothetical protein [*Vibrio *sp. 04Ya090]
5	CU052_05575	75.81	6.00E-93	gi|647,339,569|ref.|WP_025768281.1|	integrase [*Vibrio parahaemolyticus*]
5	CU052_29060	100	2.00E-107	gi|504,199,683|ref.|WP_014386785.1|	hypothetical protein [*Photobacterium damselae*]
6	CU052_00925	50.69	0.00E+ 00	gi|359,344,454|gb|AEV37828.1|	Polyketide synthase [*Pseudovibrio *sp. FO-BEG1]
6	CU052_01010	56.65	0.00E+ 00	gi|359,344,469|gb|AEV37843.1|	Polyketide synthase [*Pseudovibrio* sp. FO-BEG1]
7	CU052_28405	96.77	2.00E-80	gi|504,199,762|ref.|WP_014386864.1|	hypothetical protein [*Photobacterium damselae*]
7	CU052_28260	100	6.00E-96	gi|504,199,733|ref.|WP_014386835.1|	hypothetical protein [*Photobacterium damselae*]

### HGT candidates on chromosomes

**Fig. 6:**
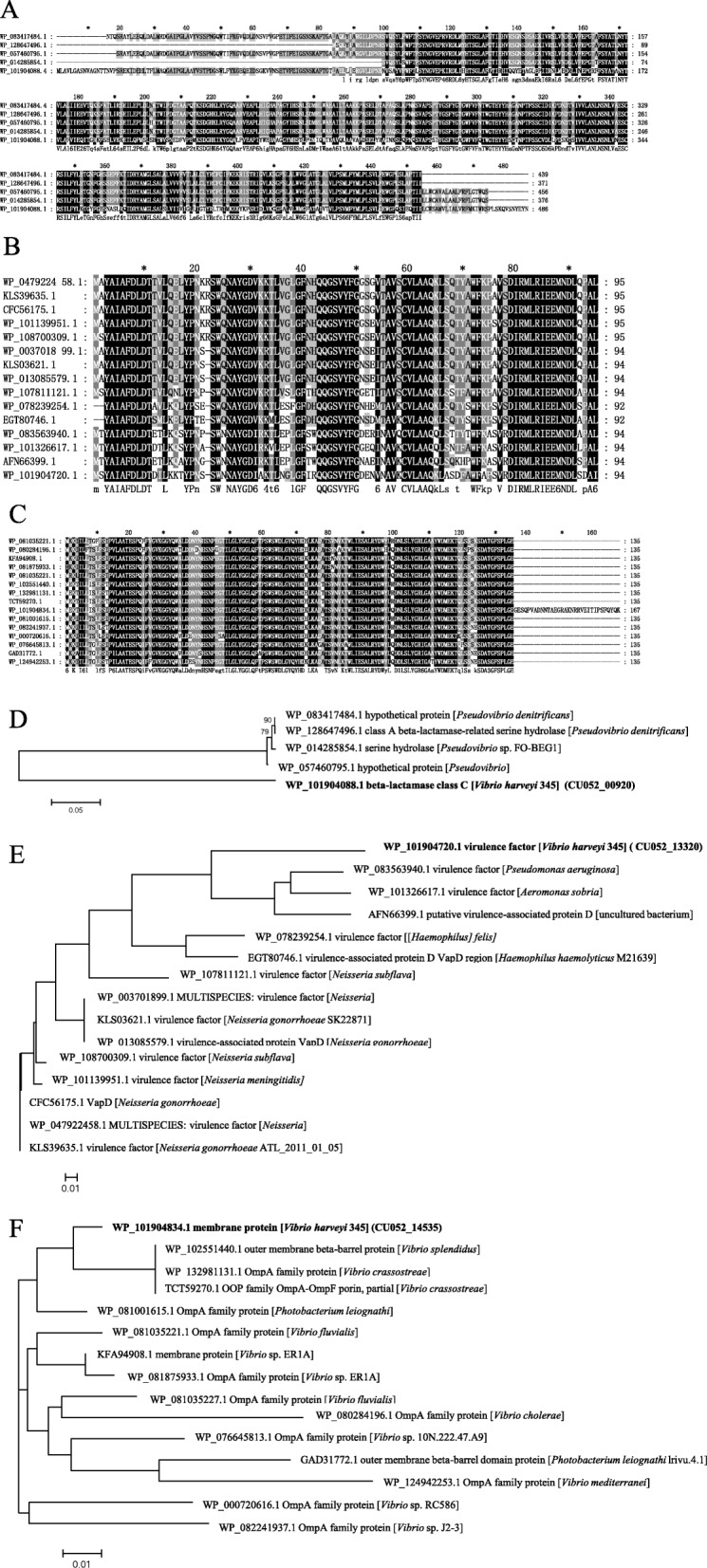
Evolutionary analysis of CU052_00920, CU052_13320, and CU052_14535. Multisequence alignment (**a**-**c**) and phylogenetic analysis (D-F) of CU052_00920, CU052_13320, and CU052_14535 with amino acids. Multisequence alignment was by ClustalW and phylogenetic tree was constructed with MEGA 6. Bar substitutions per sequence position: 0.05 (**d**), 0.01(**e**), and 0.01(**f**)

CU052_00920 in *V. harveyi* 345 encoded a beta-lactamase class C consisting of 486 amino acids. It shared no similarity with any protein of *V. harveyi*, *Vibrio* spp. or even γ-proteobacteria presently known. Instead, it shared more than 40% identity with the protein in four *Pseudovibr*io spp. (58.76–59.21% identity) suggesting recent acquisition of this gene from a *Pseudovibrio* strain (Fig. 6a and d). Two proteins were annotated, one as a serine hydrolase and the other as a class A beta-lactamase-related serine hydrolase.

CU052_13320 encoded a virulence-associated protein D of 94 amino acids. It shared 40–75.53% identity with *Pseudomonas* spp., *Aeromonas* spp., *Neisseria* spp., *Haemophilus* spp., *Rodentibacter* spp., *Alysiella* spp., *Kingella* spp., *Haemophilus* spp., and *Halomonas* spp. No similarity with any *V. harveyi* or known *Vibrio* spp. proteins was detected (data not shown). The top 15 similar strains were selected for muti-alignment and phylogenetic analysis (Fig. 6b and e). CU052_13320 was closest to a virulence factor in *Pseudomonas aeruginosa* (73.40% identity) and *Aeromonas sobria* (75.53% identity), suggesting HGT of this gene from *P. aeruginosa* or *A. sobria* (Fig. 6b and e)*.*

The CU052_14535 gene, encoding a 167 amino acid membrane protein in *V. harveyi* 345, was identified as an OmpA family protein in many other homologs. CU052_14535 showed more than 40% identity with many strains of *Photobacterium* spp., *Salinivibrio* spp., *Aeromonas* spp., *Ferrimonas* spp., *Grimontia* spp., *Aliivibrio* spp., and other *Vibrio* species, but not with *V. harveyi* (data not shown). The top 15 similar strains were selected for muti-alignment and phylogenetic analysis (Fig. 6c and f). CU052_14535 was close to an outer membrane beta-barrel protein in *V. splendidu*s (97.04% identity), and an OmpA family protein in *V. crassostreae* (97.04% identity), suggesting recent acquisition of this gene from *V. splendidu*s or *V. crassostreae* (Fig. 6c and f).

### HGT candidates on plasmids

**Fig. 7:**
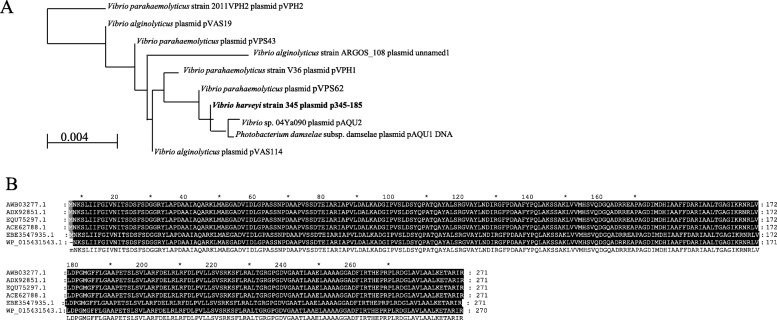
Evolutionary analysis of p345–185 and CU052_29140. Nucletides phylogenetic analysis of p345–185 (**a**) and amino acids multisequence alignment of CU052_29140 (**b**). Phylogenetic tree was constructed with the neighbor-joining method, max seq difference = 0.75 using BLAST pairwise alignments. Multisequence alignment was by ClustalW

Five antibiotic resistance genes and one virulence gene, CU052_28670, involved in immune evasion were detected on plasmid p345–185. Basic local alignment search tool (BLAST) of nucleotide (Blastn) results showed high homolog (query cover > 40% and identity > 40%) of p345–185 with plasmids pVPS62, V36, pVPS114, pVPH2, and pVPS91 from *V. parahaemolyticus*; pVAS114 and pVAS19 from *V. alginolyticus*; and pAQU1 from *Photobacterium damselae,* but no homology with other *V. harveyi* plasmids (Fig. 7a). Plasmid p345–185 showed 99.97% homolog (query cover = 99%) with pVPS62. CU052_29140 (*sul2*) was not found on 345 chromosomes and showed 100% amino acid identity to *sul2* in *Acinetobacter baumannii*, *Escherichia coli*, *Actinobacillus pleuropneumoniae*, *Histophilus somnim*, and *Salmonella enteric* (Fig. 7b).

### Nucleotide sequence accession numbers

The complete genome sequence of *V. harveyi* 345 was deposited in GenBank with accession numbers CP025537 (ChI), CP025538 (ChII), CP025539 (plasmid p345–185) and CP025540 (plasmid p345–67).

## Discussion

*V. harveyi* 345 was isolated and studied because of its multidrug resistance and serious virulence to *E. oanceolutus* in Shenzhen, Southern China. The complete genome sequence of *V. harveyi* 345 was determined and compared with 30 *V. harveyi* strains. *V. harveyi* 345 was assembled into two circular chromosomes and two plasmids. It had a larger genome than 27 strains other than KC13.17.5, E385, and 74F and had a consistent G + C content with the 30 others strains. Compared to the 30 *V. harveyi* strains, *V. harveyi* 345 had more predicated genes than the others strains except for ZJ0603. In total, 487 virulence genes encoding proteins in flagella, iron uptake, pili, LPS, CPS, chemotaxis and type II/III/IV/VI secretion involved in fish disease in clinics, were identified in *V. harveyi* 345. In addition, 25 ARGs including genes for resistance to aminoglycoside (*golc*, *adeb*, *emre*), penicillin (*pbp2*, *pbp1a*, *pbp1b*, *pbp2*), tetracycline (*tet34*, *tetm*, *tetb*), chloramphenicol (*adeb*, *ceob*, *mdtl*) and trimethoprim (*dfra26*) were found in *V. harveyi* 345, consistent with its resistance to streptomycin, ampicillin, tetracycline, chloramphenicol, trimethoprim, and sulfamethoxazole.

Virulence genes and antimicrobial-resistance genes are acquired by bacterial replication and by HGT. Horizontal transfer is the exchange of genetic material within species without any sexual mechanism [43]. This phenomenon is widely documented in bacteria and has a role in bacterial evolution and adaptation [43]. Comparative genomics of 31 *V. harveyi* strains was conducted to analyze HGT events by identifying strain**-**specific genes and strain**-**gene families. Considering the average gene number of 5087 for the 31 *V. harveyi* strains, the 3338 core genes represented approximately 66% of the total genome, meaning that approximately two-thirds of the genome was conserved among all strains. However, flower plots and dispensable heatmaps showed that each stain contained strain-specific genes, probably obtained by HGT. A total of 217 genes were specific to *V. harveyi* 345, which was more than other strains except for ZJ0603. This result suggested that a large number of HGT events happened in 345. In addition, seven gene families were specific to *V. harveyi* 345. Most (80.65%, 175/217) of the strain**-**specific genes had > 40% identify to other *Vibrio* species. The remaining came from other genera such as *Shewanella*, *Photobacterium*, *Pseudovibrio*, and *Escherichia*. We focused on the characterization of three strain**-**specific genes, CU052_00920, CU052_13320, and CU052_14535. CU052_00920 encoded a class C beta-lactamase and was transferred from *Pseudovibrio* spp. Beta-lactamase enzymes are reported to inactivate beta-lactam antibiotics by hydrolyzing the peptide bond of the characteristic four-membered beta-lactam ring, rendering the antibiotic ineffective [44]. This result suggested that CU052_00920 contributed to antibiotic resistance, especially ampicillin resistance of *V. harveyi* 345. CU052_13320 encoded a virulence-associated protein D and closes to a virulence factor in *Pseudomonas aeruginosa* (73.40% identity) and *Aeromonas sobria* (75.53% identity). CU052_13320 probably contributed to the virulence of 345. CU052_14535 encoded an OmpA family protein and was acquired from *V. splendidu*s or *V. crassostreae*. Among pathogenic bacteria, OmpA proteins are important for pathogenesis including bacterial adhesion, invasion, or intracellular survival and evasion of host defenses or stimulation of proinflammatory cytokine production [45]. This result suggested that CU052_14535 regulates the virulence of *V. harveyi* 345. DNA can also be horizontally transmitted between bacteria through plasmids, phages or uptake of naked DNA from environment [46]. Homology analysis showed that p345–185 had 99.97% homology (query cover = 99%) to pVPS62 in *V. parahaemolyticus.* PVPS62 is reported to be a pAQU-type plasmid and emerge MDR conjugative plasmid among important pathogens [47]. Five antimicrobial-resistance genes (*tetm*, *tetb*, *qnrs*, *dfra17*, *and sul2*) involved in resistance to tetracycline, fluoroquinolone, trimethoprim, and sulfonamide, and one virulence gene (CU052_28670) involved in immune evasion were located on plasmid p345–185. These genes could be horizontally transferred, promoting drug resistance and virulence. Especially, CU052_29140 (*sul2*) showing 100% amino acid identity to *sul2* in *Acinetobacter baumannii*, *Escherichia coli*, *Actinobacillus pleuropneumoniae*, *Histophilus somnim*, and *Salmonella enteric*, should contribute to the resistance of pediatric compound sulfamethoxazole tablets and sulfamethoxazole. Similarly, Klein et al. [48] found that chloramphenicol, oxytetracycline and chlortetracycline could be successful transferred by R-plasmids.

Except for plasmids, HGT encompasses a variety of genetic units, collectively known as MGEs [49] and including phages, GIs, and integrating conjugative elements (ICEs). A total of 47 GIs, with encoding many transposases, integrases, and recombinases, and two incomplete prophage sequences were identified in *V. harveyi* 345. These genetic units probably acted as HGT delivery tools, contributing to pathogenesis, drug resistance and environmental adaptation of *V. harveyi* 345. Several type III/VI secretion proteins along with IS family transposases were predicted on the same GI that could be easily transmitted. Dissemination of these genes could further compromise *Vibrio* infections, limiting treatment options. These results further indicated that HGT contributed to virulence and antibiotic resistance of *V. harveyi* 345.

The 345 strain was isolated from a diseased *E. oanceolutus* in Shenzhen, Southern China. Recent increasing aquaculture density has led to frequent breeding diseases and increased use of antibiotics in Southern China, resulting in serious antibiotic residues and pollution [50]. Intensification of coastal urbanization has led to massive discharge of pollutants (heavy metals, nutrients, biocides), and intensification of human activities has caused environmental climate change, especially global warming [51]. Many studies show that the wide use of antibiotics; pollution by heavy metals, nutrients and biocides; and global climate change regulate HGT by affecting plasmid replication, changing phage activity, adjusting HGT**-**related enzyme activity, and damaging immune systems. These factors regulate bacterial resistance and pathogenicity [52–54]. For example, Beaber et al. [53] found that ciprofloxacin induces transfer of SXT, an ICE derived from *V. cholera* encoding genes that confer resistance to chloramphenicol, sulphamethoxazole, trimethoprim and streptomycin. The mechanism is increasing expression of SXT activators, enhancing drug resistance. Similarly, high environmental temperatures in combination with UV irradiation accelerate the spread of *stx* (Shiga toxin) genes by enhancing Stx prophage induction and Stx phage-mediated gene transfer [52]. Therefore, environmental pollution and climate change may increase the communication of virulence genes and drug resistance genes by affecting HGT. The result will enhance the toxicity and drug resistance of *V. harveyi* and affect the ecological safety of aquaculture ecosystems.

## Conclusions

We present the first complete genome of the serious disease-causing *V. harveyi* 345 which has a multidrug-resistant phenotype. We did comparative genomics between this strain and 30 other *V. harveyi* strains. The genome was determined to study *V. harveyi* 345 virulence and antimicrobial resistance. Multiple virulence factors and resistance genes were predicted in this strain, consistent with results of Jiang et al. [38]. We searched for evidence of HGT and evaluated genomic traits relating to HGT. The high quality complete genome sequence generated in this study will aid further studies for a deeper understanding of the mechanisms of *Vibrio* pathogenesis and antibiotic resistance. These studies could improve seafood quality and reduce economic loss. We recommend that more research be done on the response mechanism of HGT relative to environmental change and antibiotic use. This research will be an important scientific basis for predicting outbreaks and controlling *V. harveyi* disease. As environmental pollution including use of antibiotics and climate change enhance the virulence and drug resistance of pathogens, research should explore management approaches, for example by bacteriophages, to manage the occurrence of *V. haveyi* in the environment [55].

## Methods

### DNA extraction

*V. harveyi* 345 was grown overnight in 2216E medium (BD, USA) at 28 °C with vigorous shaking. Overnight cultures were inoculated with a 1:1000 dilution into 20 ml fresh 2216E medium and grown until OD600 = 0.5. Cells were collected by centrifugation and genomic DNA extracted using the CTAB method [56]. The quantity and quality of genomic DNA were evaluated using a Qubit Fluorometer (Invitrogen, Carlsbad, CA, USA) and 1% agarose gel electrophoresis. DNA was stored at − 80 °C and prepared for genome sequencing.

### Whole genome sequencing and assembly

A PacBio RSII 20-kb template library was constructed from at least 10 μg genomic DNA at the Beijing Genomics Institute (BGI). The library was subjected to quality control (QC) with Qubit Fluorometer (Invitrogen, Carlsbad, CA, USA) and Nanodrop 2000 (Thermo Fisher Scientific, Wilmington, DE, USA) to check the concentration, purity and integrity of library templates. Libraries were sequenced on Illumina Hiseq 4000 and Pacbio RSII sequencing platforms. Genomes were primarily assembled with a variety of software for short sequence assembly based on clean data from the Illumina Hiseq 4000 platform. RS_HGAP Assembly3 of SMRT Analysis v.2.3.0 was used to assemble data from the Pacbio RSII platform. Contigs were error corrected successively by soapSNP and soapIndel software and GATK analysis. Gaps between contigs were filled by PCR.

### General annotation

ORFs were predicted by Glimmer v.3.02 [57], and genome annotation was completed using the NCBI prokaryotic genome automatic annotation pipeline with identity ≥40% and E_value ≤10^− 5^ (Blast and annotation for all study sets used the same identities and E_values). COG, KEGG, GO and NR databases were used to search domain architecture. The tRNA genes were identified by tRNAscan-SE v.1.23 [58] and rRNA genes with RNAmmer v.1.2 [59]. The sRNAs were predicted by blasting nucleic acid against Rfam database [60]. Tandem repeats were predicted by Tandem Repeat Finder v.4.04 [61].

### Identification of virulence factors and ARGs

Putative virulence factors of *V. harveyi* 345 were predicted by blasting against the Virulence Factor Database (VFDB) (http://www.mgc.ac.cn/VFs/main.htm) [62] and ARGs by blasting against the Antibiotic Resistance Genes Database (ARDB) (http://ardb.cbcb.umd.edu/) [63].

### Prediction of GIs, prophages, and CRISPR-Cas systems

GIs, prophages, and CRISPR-Cas systems were respectively identified using online tools IslandViewer 4, PHAge Search Tool (PHAST), and CRISPR Finder software [64–66].

### Comparative genomics

Comparative genome analysis was conducted among 31 *V. harveyi* strains with *V. harveyi* VH2 as the reference strain and an average gene number of 5087 (Additional file 2: Table S2) to assess *V. harveyi* evolution. A phylogenetic tree was constructed based on the single nucleotide polymorphism (SNP) matrix of the 31 strains with the maximum-likelihood method, bootstrapped 1000 times via Treebest-1.9.2 software [67].

### Extraction of pan-, core-, dispensable and strain-specific genes

Core-pan genes were analyzed using a BLAST method [68]: Genes were taken from reference genome *V. harveyi* VH2 as a gene pool. Genes predicted in one strain among other 30 strains (Query samples) were BLAST with the gene pool, and results filtered by length and identity (> 40%). BLAST coverage ratios (BCR, reference BCR = match/reference length × 100%, query BCR = match/query length × 100%, and match stands for the length used for BLAST) of genes from the gene pool and Query samples were calculated separately. If BCR values from the reference and Query sample were smaller than the setting value (40%), the reference gene did not have homology with the Query gene. The nonhomologous gene from the Query genome was added to the gene pool. Query samples were processed and the final gene pool used as the pan gene pool. The non-homolog genes from the reference genome were the strain specific genes against the Query sample. Nonhomologous genes were BLAST with the gene pool of another Query sample, repeated for all samples, and the final nonhomologous gene pool was used as the strain-specific gene pool.

After aligning to genes from samples, BCR values of genes from the pan gene pool were calculated for each sample. The coverage array was generated for this pool. If the BCR value of a gene in each sample was larger than the setting value, the gene was a core gene. If the gene was predicted from an assembled result, BLAST results were filtered and the sequence was removed if the number N (N means uncertain nucleotides in gene) was larger than the setting (30%) for the gene. Dispensable genes were included in the pan-gene pool but not the core-gene pool.

### Construction of gene families

Gene families were constructed using genes from the reference strain and the target bacterium. Protein sequences were aligned with basic local alignment search tool of protein (Blastp) and redundancy was eliminated. Gene family TreeFam clustering was carried out with alignment results and Hcluster_sg software. Alignment results for proteins were converted into multiple sequence amino acids in the CDS area after multiple sequence alignments with the clustered gene family using Muscle software [69]. Gene family tree construction analysis was carried out for multiple sequence alignment results using Muscle and neighborhood-joining (NJ) method with Treebest software [67].

### Characterization of putative HGT

Of the strain-specific genes, we extracted CU052_00920, encoding a beta-lactamase class C; CU052_13320, encoding a virulence-associated protein D; and CU052_14535 encoding an OmpA family protein for further analysis on contribution to the antibiotic resistance or virulence of *V. harveyi* 345. The genes were compared to the NR protein database of NCBI using the Blastp tool. The results were filtered manually to find significant hits to proteins belonging to species other than *V. harveyi*. Multiple sequence alignment was conducted using ClustalW [70]. An alignment phylogenetic tree was constructed from aligned sequences using the Kimura 2-parameter model [71] with the neighbor-joining method, bootstrapped 1000 times, with MEGA6.0 software [72].

In addition, the entire sequence of p345–185 was compared to the NR nucleotide database of NCBI using a Blastn tool. A phylogenetic tree was constructed with the neighbor-joining method using max seq difference = 0.75 and BLAST pairwise alignments. The characterization method was the same for CU052_29140 (*sul2*) on p345–185 as for CU052_00920, CU052_13320, and CU052_14535.

## Supplementary information

**Additional file 1: Table S1.** Global comparison of pollutant (antibiotics, heave metals, and nutrients) concentrations of water samples.

**Additional file 2: Table S2.** Gene information and gene statistics for 31 *V. harveyi* strains.

**Additional file 3: Figure S1.** KEGG pathway classifications of *V. harveyi* 345 coding sequences.

**Additional file 4: Figure S2.** GO functional classifications of *V. harveyi* 345 coding sequences.

**Additional file 5: Table S3.** Details of virulence factors predicted with the VFDB database.

**Additional file 6: Table S4.** Genomic islands in a single chromosome (CP025537) of *V. harveyi* 345.

**Additional file 7: Table S5.** Genomic islands in a single chromosome (CP025538) of *V. harveyi* 345.

**Additional file 8: Table S6.** Prophage sequences present in *V. harveyi* 345 chromosomes.

**Additional file 9: Table S7.** Strain-specific genes of *V. harveyi* 345.

## Data Availability

Data generated or analysed during this study are included in this published article and its supplementary information files. Genomic data are available from the GenBank (accession numbers: CP025537, CP025538, CP025539 and CP025540).

## Abbreviations

ARDB: Antibiotic resistance genes database
ATG: Antibiotic-resistance gene
BGI: Beijing Genomics Institute
BLAST: Basic local alignment search tool
COG: Clusters of orthologous groups
GIs: Genetic islands
GO: Gene ontology
HGT: Horizontal gene transfer
ICEs: Integrative and conjugative elements
KEGG: Kyoto encyclopedia of genes and genomes
LD50: Median lethal dose
MDR: Multidrug resistance
MGE: Mobile genetic element
NJ: Neighborhood-joining
NR: Non-Redundant
ORFs: Open reading frames
PHAST: PHAge Search Tool
QC: Quality control
RM: restriction modification
SNP: Single nucleotide polymorphism
T6SS: Type VI secretion system
TLH: Thermolabile hemolysin
VFDB: Virulence factor database
