# Supplementary material for: Horizontal gene transfer contributes to virulence and antibiotic resistance of Vibrio harveyi 345 based on complete genome sequence analysis
Source: BMC Genomics. 2019 Oct 22;20:761. doi: 10.1186/s12864-019-6137-8 (PMC6805501; doi:10.1186/s12864-019-6137-8)
Supplement: Supplementary file 1 — Additional file 1: Table S1. Global comparison of pollutant (antibiotics, heave metals, and nutrients) concentrations of water samples. [file 12864_2019_6137_MOESM1_ESM.doc]

**Table S1** Global comparison of pollution (antibiotics, heave metals, and nutrients) concentrations of water samples

| Pollution types | Pollutions | Sampling locations | | | | |
| --- | --- | --- | --- | --- | --- | --- |
|  |  | Guangdong costal | Po River | Rivers of Madrid downstream | River Tyne | Surface waters |
|  |  | China | Italy | Spain | United Kingdom | Germany |
| Antibiotics | Sulfadiazine | 3.145 | - | - |  | - |
| (ng/L) | Sulfadimethylpyrimidine | 15.700 | - | - |  | - |
|  | Sulfamethoxazole | 8.545 | - | - |  | 0.480 |
|  | Norfloxacin | 68.520 | - | 10.000 |  | - |
|  | Ofloxacin | 6.930 | - | - |  | - |
|  | Tetracycline | 6.030 | - | - |  | n.d. |
|  | Anhydroerythromycin | 21.705 | - | - |  | - |
|  | Enrofloxacin | 4.700 | - | - |  | - |
|  | Roxithromycin | 2.725 | - | - |  | 0.560 |
|  | Ciprofloxacin | 8.029 | - | 3.000 |  | - |
|  | Erythromycin | 529.5 | 2.950 | 320.500 | 37.000 | 1.700 |
|  | Trimethoprim | - | - | 424.000 | 11.500 | 0.200 |
|  | Clarithromycin | - | - | 235.000 |  | 0.260 |
|  | Chloramphenicol | - | - | - |  | 0.060 |
|  | **References** | [1-3] | [4] | [5] | [6] | [7] |
|  |  | Guangdong costal | Sydney Harbour | Klang Strait, | Tsurumi River |  |
|  |  | China | Australia | Malaysia | Japan |  |
| Heavy metals | Cr | 33.000 | - | 4.504 | - |  |
| (μg/L) | Ni | 4.000 | 0.860 | 2.363 | - |  |
|  | Cu | 11.000 | 1.680 | 2.508 | - |  |
|  | Mn | 68.000 | 20.000 | - | - |  |
|  | Zn | 48.000 | 6.470 | 50.833 | - |  |
|  | Cd | 288.000 | 0.040 | 0.493 | 5.000 |  |
|  | Pb | 28.000 | - | 3.819 | 7.000 |  |
|  | Hg | 326.000 | - | 0.024 | - |  |
|  | Fe | 889.000 | - | - | - |  |
|  | As | 378.000 | - | 25.348 | 6.000 |  |
|  | Co | - | - | - | 50.000 |  |
|  | Sr | - | - | - | 208.000 |  |
|  | **References** | [8] | [9] | [10] | [11] |  |
|  |  | Guangdong costal | Tokyo Bay | Kaneohe Bay | Alfacs Bay | Paranaguá Bay |
|  |  | China | Japan | Hawaii | NW Mediterranean | Brazil |
| Nutrients | DIN | 1.898 | 0.700 | 0.024 | 0.560_max_ | 0.153 |
| (mg/L) | DIP | 0.266 | 0.030 | 0.007 | 0.047_max_ | 0.014 |
|  | **References** | [12] | [13] | [14] | [15] | [16] |

Note: -, not analyzed; n.d., not detected; DIN, including NO_2_^-^-N、NO_3_^-^-N and NH_4_^+^-N; DIP, including PO_4_^3-^-P; all the concentrations have been shown as the average concentrations, except the nutrients concentration in Alfacs Bay, NW Mediterranean.

References

1. Liang X, Chen B, Nie X, Shi Z, Huang X, Li X. The distribution and partitioning of common antibiotics in water and sediment of the Pearl River Estuary, South China. Chemosphere. 2013;92:1410-1416.
2. Xu WH, Yan W, Li XD, Zou YD, Chen XD, Huang WX, et al. Antibiotics in riverine runoff of the Pearl River Delta and Pearl River Estuary, China: Concentrations, mass loading and ecological risks. Environ Pollut. 2013;182:402-407.
3. Xu WH, Zhang G, Zou SC, Li XD, Liu YC. Determination of selected antibiotics in the Victoria Harbour and the Pearl River, South China using high-performance liquid chromatography-electrospray ionization tandem mass spectrometry. Environ Pollut. 2007;145:672-679.
4. Calamari D, Zuccato E, Castiglioni S, Bagnati R, Fanelli R. Strategic survey of therapeutic drugs in the rivers Po and Lambro in northern Italy. Environ Sci Technol. 2016;37:1241-1248.
5. Valcárcel Y, Alonso SG, Rodríguez-Gil JL, Gil A, Catalá M. Detection of pharmaceutically active compounds in the rivers and tap water of the Madrid Region (Spain) and potential ecotoxicological risk. Chemosphere. 2011;84:1336-1348.
6. Roberts PH, Thomas KV. The occurrence of selected pharmaceuticals in wastewater effluent and surface waters of the lower Tyne catchment. Sci Total Environ. 2006;356:143-153.
7. Hirsch R, Ternes T, Haberer K, Kratz KL. Occurrence of antibiotics in the aquatic environment. Sci Total Environ. 1999;225:109-118.
8. Xie W, Chen K, Zhu X, Nie X, Zheng G, Pan D. Evaluation of heavy metal contents in water and fishes collected from the waterway in Pearl River Delta in south China. J Agro-Environ Sci. 2010;29:1917-1923.
9. Hatje V, Apte SC, Hales LT, Birch GF. Dissolved trace metal distributions in Port Jackson estuary (Sydney Harbour), Australia. Mar Pollut Bull. 2003;46: 719-730.
10. Sany SBT, Salleh A, Sulaiman AH, Sasekumar A, Rezayi M, Tehrani GM. Heavy metal contamination in water and sediment of the Port Klang coastal area, Selangor, Malaysia. Environ Earth Sci. 2013;69.
11. Mohiuddin KM, Otomo K, Ogawa Y, Shikazono N. Seasonal and spatial distribution of trace elements in the water and sediments of the Tsurumi river in Japan. Environ Monit Assess. 2012;184:265-279.
12. Zhang J, Zhang Y, Zhou K, Zhang J, Li X. Evaluation on temporal and spatial distribution of nutrients and potential eutrophication in Shenzhen Bay. Ecol Environ Sci. 2010;19:253-261.
13. Kodama K, Oyama M, Lee JH, Kume G, Yamaguchi A, Shibata Y, et al. Drastic and synchronous changes in megabenthic community structure concurrent with environmental variations in a eutrophic coastal bay. Prog Oceanogr. 2010;87: 157-167.
14. Drupp P, Carlo EHD, Mackenzie FT, Bienfang P, Sabine CL. Nutrient inputs, phytoplankton response, and CO_2_ variations in a semi-enclosed subtropical embayment, Kaneohe Bay, Hawaii. Aquat Geochem. 2011;17:473-498.
15. Llebot C, Spitz YH, Solé J, Estrada M. The role of inorganic nutrients and dissolved organic phosphorus in the phytoplankton dynamics of a Mediterranean bay : A modeling study. J Marine Syst. 2010;83:192-209.
16. Mizerkowski BD, Ladwig N, Machado EDC, Rosa R, Araujo T, Koch D,. Sources, loads and dispersion of dissolved inorganic nutrients in Paranaguá Bay. Ocean Dynam. 2012;62:1409-1424.
